# Salinization influences the biodiversity‐ecosystem functioning relationship more strongly at high salinity

**DOI:** 10.1002/mlf2.70034

**Published:** 2025-11-18

**Authors:** Jianrong Huang, Mingxian Han, Jian Yang, Yi Wang, Hongchen Jiang

**Affiliations:** ^1^ School of Life Sciences Henan University Kaifeng China; ^2^ Qinghai Provincial Key Laboratory of Geology and Environment of Salt Lakes, Qinghai Institute of Salt Lakes Chinese Academy of Sciences Xining China; ^3^ School of Mathematics and Physics China University of Geosciences Wuhan China

**Keywords:** community assembly, environmental filtering, functional redundancy, microbial diversity, osmotic stress

## Abstract

Salinization threatens ecosystem stability by altering microbial diversity and function, yet how salinity influences biodiversity‐ecosystem functioning (BEF) relationships remains unclear. In this study, we constructed artificial microbial communities (5–40 strains) with varying salt tolerance and phylogeny, culturing them across a salinity gradient (0.9%–20%). We found that low‐to‐moderate salinity (0.9%–7%) minimally affected BEF relationships, but high salinity (15%–20%) amplified biodiversity loss impacts, with ecosystem yield declining sharply at low richness (*R* ^2^ = 0.573, *p* < 0.001). Hypersaline conditions shifted community composition toward halophiles (e.g., *Halomonas* dominance at 20% salinity) and inhibited metabolic functions, such as glycosyl hydrolase activity. Mechanistically, selection effects predominated at 15% salinity (contributing 63.1%–72.0% to net biodiversity effects), whereas complementarity effects were diminished. These findings underscore the vital role of biodiversity in mitigating hypersaline stress and highlight the necessity for targeted strategies to enhance ecosystem resilience against global salinization.

## Impact statement

The impact of salinization on biodiversity and ecosystem functioning (BEF) is not well understood across different salinity levels. Our study shows that as salinity increases, the BEF relationships change, with minor effects at low salinity but significant declines in high salinity areas. Biodiversity loss harms ecosystem functioning under high salinity due to increased osmotic stress, which elevates species interdependence. As salinity rises, the importance of selection effects increases, while complementary effects decline. Thus, more species are needed to maintain ecosystem functioning in high salinity, highlighting biodiversity's crucial role in ensuring ecosystem stability under environmental stresses.

## INTRODUCTION

Salinization, driven by climate change and human activities, significantly impacts ecosystems globally^1^, ^2^, ^3^, ^4^. The most immediate result is an increase in salinity^5^, which can hinder growth, reduce biodiversity, and alter community composition and metabolic functions^6^, ^7^, ^8^, ^9^. For example, higher salinity leads to a linear decrease in soil microbial alpha diversity and reduces enzyme activity related to carbon uptake and sediment denitrification^10^, ^11^, ^12^, ^13^. While previous research has typically examined microbial diversity and function independently, they often overlook how the loss of microbial diversity from salinization affects overall ecosystem function^14^, ^15^, ^16^, ^17^, ^18^. The biodiversity‐ecosystem functioning (BEF) relationship is critical in ecology, as studies show that ecosystems with higher biodiversity tend to be more productive and stable^15^, ^19^, ^20^. However, recent findings indicate that environmental stresses, like salinity, can disrupt this relationship. A recent study noted that the impact of microalgae biodiversity on ecosystem function diminished with increasing salinity (0–2.5%)^21^. However, there is still a limited understanding of how variations in biodiversity due to salinity changes affect ecosystem functions across a broader salinity range.

Two fundamental processes, selection effects and complementarity effects, have been proposed to elucidate the intricate relationship between biodiversity and ecosystem functioning^22^. Selection effects arise from mechanisms such as interspecific competition, resulting in the predominance of species endowed with advantageous traits, thereby positively influencing ecosystem productivity. In contrast, complementarity effects pertain to niche differentiation and the interplay of interspecific promotion or inhibition, which can enhance the performance of individual species and elevate resource use efficiency and productivity within more diverse communities^22^, ^23^, ^24^, ^25^, ^26^. Evaluating the relative significance of these two foundational processes is essential for comprehending how ecosystems adapt to changes in biodiversity and for informing the management of natural ecosystems^27^. Various studies have indicated that selection effects predominantly govern the impact of bacterial diversity on community biomass^28^, overall community productivity, and specific microbial functions, such as β‐glucan degradation^29^. Conversely, other research posits that complementarity effects are indispensable to understanding the dynamics between biodiversity and ecosystem function, influencing factors including the overall bacterial density linked to bioaugmentation^27^, as well as denitrification and respiration processes^30^. The influences of biodiversity on ecosystem function are well known, particularly evident amid environmental shifts, such as drought, warming, and variations in salinity^21^, ^24^, ^31^, ^32^. Nevertheless, there remains a paucity of information regarding how the relative contributions of selection and complementarity effects to the BEF nexus are affected by escalating salinity levels.

In this study, we hypothesize that (1) the increase of salinity may significantly alter the relationship between biodiversity and ecosystem functioning and (2) concerning the interplay between microbial biodiversity and salinity in shaping ecosystem functions, we anticipate that the explanatory power of selection effects may amplify with rising salinity, while the influence of complementarity effects may diminish. To test these hypotheses, we constructed artificial microbial communities (5, 10, 20, and 40 strains) with varying salt tolerance and phylogeny, culturing them across a salinity gradient (0.9%, 3.5%, 7%, 15%, and 20%). To assess how salinity fluctuations affect BEF relationships through microbial species loss, we systematically quantified community biomass dynamics. In the initially homogenized 40‐species consortium (highest richness), we further tracked salinity‐induced shifts in community composition and metabolic functions, including enzymatic activities and carbon substrate utilization patterns.

## RESULTS

### The strength of the BEF relationship under varying salinities

**Figure 1:**
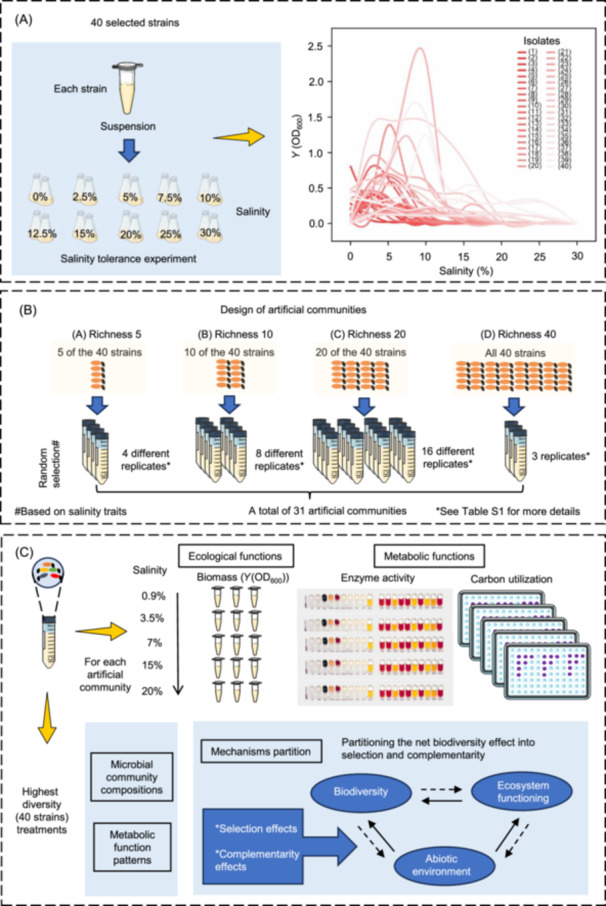
Schematic representation of the experimental setup in this study. (A) Salt tolerance curves for the 40 isolates generated by measuring the average optical density (OD_600_) of each strain across 10 salinity levels. (B) Design of artificial communities with different species richness (5, 10, 20, and 40 strains). (C) Assessing of ecological/metabolic functions of all assembled artificial communities. The community with the highest diversity (40 strains) was used to analyze composition and function changes, and the biodiversity effects were partitioned into selection and complementarity effects. *Y*, biomass yield.

**Figure 2:**
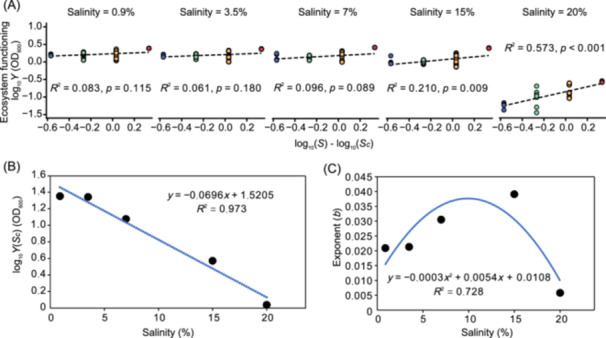
Effects of salinity on species richness and ecosystem functioning relationships. (A) Effects of salinity on the relationship between species richness and ecosystem functioning. (B) The intercept of the diversity‐functioning relationship declining with increasing salinity. (C) Changes in the exponent revealing a weakly inverted U‐shaped relationship with salinity. *S*, species richness; *S_C_*, mean‐centered species richness.

Across the experimental salinity gradients ranging from 0.9% to 20%, ecosystem functioning—quantified through community biomass yield—demonstrated distinct responses to variations in species richness that depended on the intensity of salinity stress (Figure 1). Specifically, artificial communities subjected to low‐to‐moderate salinities (0.9%, 3.5%, and 7%) exhibited consistently high levels of ecosystem functioning, regardless of changes in species richness (*p* > 0.05). In stark contrast, under hypersaline conditions (15% and 20%), ecosystem functioning increased significantly with rising species richness, as indicated by stronger positive correlations (15%: *R* ^2^ = 0.210, *p* = 0.009; 20%: *R* ^2^ = 0.573, *p* < 0.001; Figure 2A). Furthermore, the intercept of the BEF relationship, reflecting the average community yield at intermediate levels of species richness, was found to decline linearly with increasing salinity (*R* ^2^ = 0.973, *p* < 0.01; Figure 2B). This suggests that, on average, communities in more saline environments supported lower levels of asymptotic biomass. Additionally, salinity notably influenced the exponent (b) of the relationship between species richness and ecosystem functioning (Figure 2C). While an increase in salinity from 0.9% to 15% strengthened the biodiversity effects (i.e., enhanced the exponent), a pronounced reversal occurred at 20% salinity, where the linkage between diversity and ecosystem functioning substantially weakened. This indicates that high salinity levels disrupt the positive contributions of higher diversity to ecosystem functioning, underscoring the complexity of the BEF relationship in response to environmental stresses.

We also examined the influence of species richness on metabolic functions, such as enzyme activity and carbon utilization, across the established salinity gradients (Figure S1). Remarkably, the patterns observed in metabolic functions closely resembled those seen in overall ecosystem functioning. Specifically, artificial communities subjected to low‐to‐moderate salinities (0.9%, 3.5%, and 7%) consistently exhibited high levels of enzymatic activity, regardless of variations in species richness. However, under the more extreme hypersaline conditions (15% and 20%), we observed a significant increase in both enzyme activity and carbon source utilization as species richness rose (*p* < 0.05; Figure S1). This divergence underscores the critical role of biodiversity in promoting metabolic performance, highlighting that the benefits of increased species richness are particularly pronounced under conditions of acute salinity stress. Thus, while low salinity environments may support high metabolic activity across varying levels of biodiversity, it is under high salinity stress that the contribution of biodiversity to metabolic functions becomes particularly essential.

### Variations of community compositions and metabolic function patterns under varying salinities

**Figure 3:**
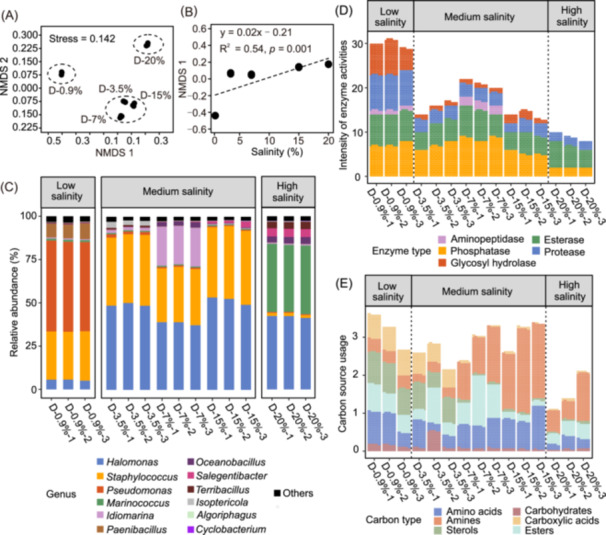
Microbial compositional turnover associated with variance in salt tolerance traits of the strains. (A) Nonmetric multidimensional scaling analysis (NMDS) of the microbial communities in the highest diversity treatments (Group D, richness = 40). (B) Relationship between species scores extracted from NMDS1 and salinity. (C) Variations in microbial community composition in the highest diversity treatments. (D, E) The intensity of five types of enzyme activities (D) and the carbon utilization pattern of six types of carbon sources (E) in the highest diversity treatments.

To better understand the effects of salinity on community composition and metabolic functions, we investigated a high‐diversity microbial consortium composed of 40 species, which was initially homogenized across replicate samples (Figure 1C). This consortium was cultivated under five different salinity regimes, ranging from physiological saline (0.9%) to hypersaline levels (20%). The results of the nonmetric multidimensional scaling (NMDS) analysis revealed clear separation of the microbial consortium's composition based on the different salinity levels, forming three distinct clusters (Figure 3A). To facilitate further differentiation and comparison, the community compositions were categorized into three groups: low salinity, medium salinity, and high salinity. A one‐way permutational multivariate analysis of variance (PERMANOVA) confirmed that the compositions varied significantly with salinity (*R* ^2^ = 0.92, *p* = 0.001), as detailed in Table S1, which includes pairwise comparisons. Moreover, species scores obtained from the primary axis of the NMDS (NMDS1) exhibited a significant positive correlation with salinity (*R* ^2^ = 0.54, *p* = 0.001; Figure 3B), indicating that salinity plays a crucial role in determining community structure. The dominant genera within the microbial consortium also varied significantly among the different salinity levels (Figure 3C). At the lowest salinity level (0.9%), the dominant genera included *Pseudomonas*, *Staphylococcus*, and *Paenibacillus*. As salinity increased to 3.5%, 7%, and 15%, the dominant genera shifted to *Halomonas*, *Staphylococcus*, and *Idiomarina*, respectively. At the highest salinity level of 20%, the consortium was primarily composed of *Halomonas*, *Marinococcus*, *Oceanobacillus*, and *Salegentibacter*.

The metabolic functions of the microbial consortium varied significantly across the five different salinities. Notably, with respect to enzyme activities, we observed that the intensity of glycosyl hydrolases in microbial communities cultured at salinities of 3.5%, 7%, and 15% was lower than that at 0.9% salinity, and glycosyl hydrolase activity was completely absent at 20% salinity. This suggests that increasing salinity negatively impacts the activity of these enzymes, which are crucial for carbohydrate metabolism. Similarly, the protease activity exhibited a clear pattern: the microbial communities cultivated at 0.9% salinity demonstrated higher protease intensity compared to those grown at higher salinities (Figure 3D). This indicates that lower salinity environments are more conducive to protease activities, which are important for protein degradation and utilization. In terms of carbon source utilization, the microbial communities at salinities of 0.9% and 3.5% showed a preference for utilizing carboxylic acids and sterols; however, this utilization decreased markedly as salinity increased to 7%, 15%, and 20%. Conversely, the utilization of amines by these communities increased with higher salinity levels (Figure 3E). This trend suggests a shift in metabolic strategies as salinity rises, with microbial communities adapting to utilize different carbon sources that might be more accessible under high salinity conditions.

### Selection and complementarity effects

**Figure 4:**
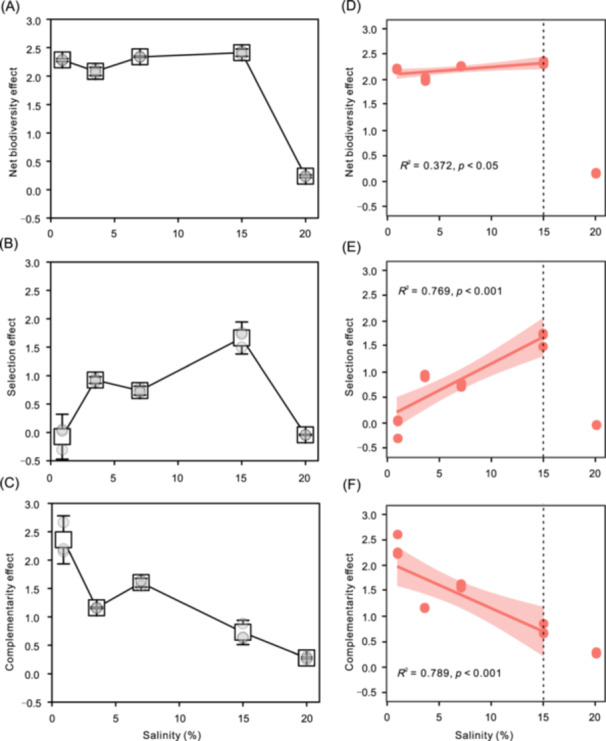
Partitioning the effects of salinization on ecosystem function (biomass density) into selection and complementarity effects. (A–C) Net biodiversity effect (NBE) (A), selection effect (B), and complementarity effect (C) of biodiversity on function at each salinity treatment. (D–F) Linear relationship between salinity (within the 0–15% range) and NBE (D), selection effect (E), and complementarity effect (F). Red shading indicates 95% confidence.

To investigate the mechanisms through which species loss and salinity collectively influence ecosystem functioning, we analyzed the net biodiversity effect (NBE) by breaking it down into its key components: selection and complementarity effects. The results revealed a positive NBE across all salinity treatments (values greater than 0), with the NBE being notably higher in the 0–15% salinity range compared to the 20% salinity level (Figure 4A and Table S2). Both selection effects and complementarity effects contributed to the overall NBE; however, their relative contributions varied with salinity levels (as detailed in Figure 4 and Table S2). Specifically, within the salinity range of 0–15%, we observed that the selection effect increased with rising salinity levels, suggesting that as salinity increases, the ability of certain species to thrive and exert a positive influence on ecosystem functioning becomes more pronounced. Selection effects dominated at 15% salinity, contributing 63.1%–72.0% to NBEs. In contrast, complementarity effects displayed a decreasing trend with higher salinity, indicating that the diversity of interactions that enhance resource use efficiency among species diminishes. Linear regression analysis further supported the observed correlation between selection effects and salinity within the 0–15% range, highlighting the positive relationship. However, at the extreme salinity level of 20%, the contribution of complementarity effects was minimal, suggesting that microbial ecosystem functioning is likely severely compromised when salinity surpasses the salt tolerance threshold for many species within the community. Analysis of variance (ANOVA) results indicated significant differences (*p* < 0.001) among the various effects across different salinity treatments, specifically focusing on the NBE, selection effect, and complementarity effect (Table S3). Post hoc Tukey's Honestly significant difference (Tukey's HSD) tests confirmed statistically significant pairwise comparisons between different salinity levels (*p* < 0.05), with the exception of the groups highlighted in gray, which were not significantly different (Table S4). Overall, these findings illustrate that salinity profoundly influences microbial community dynamics and ecosystem functioning, with the balance between selection effects and complementarity effects varying significantly across different salinity conditions. As salinity approaches or exceeds the tolerance limits of the microbial species involved, the beneficial contributions of biodiversity to ecosystem functioning are diminished, underscoring the critical role of salinity in shaping microbial ecosystems.

### Salinity mediates the BEF relationship

**Figure 5:**
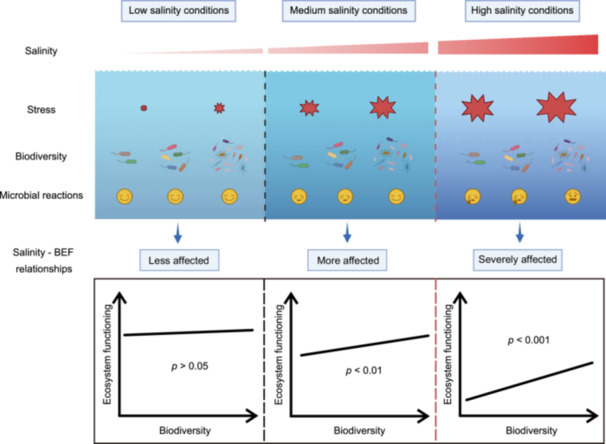
Salinity mediates the relationship between biodiversity and ecosystem functioning. Schematic diagram illustrates the hypothetical shift in relationships between biodiversity and ecosystem functioning with increasing salinity.

We developed a conceptual model to clarify how the BEF relationship responds to variations in salinity (Figure 5). Under low salinity conditions, we found that increases in salinity had minimal impact on the BEF relationship, indicating that species richness had little influence on microbial ecosystem functioning (*p* > 0.05). This suggests that at lower salinities, the microbial communities are robust and resilient, allowing them to maintain functionality despite changes in salinity. As salinity increased to medium levels, the effects began to change. While these increases did not lead to catastrophic failures in microbial functionality, we noted progressively more pronounced negative effects (*p* < 0.01). This trend indicated that a moderate rise in salinity can weaken microbial community activity, thus gradually impacting their overall functionality. Within a specific salinity range (up to 15% in this study), we observed that higher microbial diversity significantly enhanced the resilience of community structure and function against salinity stress, thereby stabilizing ecosystem performance. However, exceeding a critical threshold (e.g., 20% salinity in this study) resulted in severe impairment of microbial ecosystem functioning, potentially leading to a complete loss of function and the inability to recover. Interestingly, even under these extreme conditions, greater microbial biodiversity was shown to positively influence ecosystem functioning, providing some level of mitigation against the decline in community performance. These findings highlight salinization as a critical factor that shapes the balance between biodiversity and ecosystem functioning. This relationship is especially relevant in the context of global climate change and environmental shifts, as increasing salinity levels are likely to affect microbial communities and their contributions to ecosystem processes. Overall, the model emphasizes the importance of maintaining biodiversity to enhance resilience against salinity stress and sustain ecosystem functioning amidst changing environmental conditions.

## DISCUSSION

### The synergistic effects of salinity and biodiversity on ecosystem functions

Our findings illustrate that changes in salinity systematically influence BEF relationships, with varied responses observed across different salinity ranges (Figure 2A). At low salinities (0.9%, 3.5%, and 7%), the impact of salinity on the BEF relationships was relatively minimal (*p* > 0.05), indicating that osmotic interference was not a significant concern in this range. This suggests that microbial communities are largely unaffected by salinity at these lower levels, allowing for stable ecosystem functioning despite variations in biodiversity. In contrast, as salinity increased to higher levels (specifically at 15% and 20%), we observed a significant negative impact on the BEF relationship (*p* < 0.01). This elevation in salinity exacerbated the functional consequences resulting from biodiversity loss, indicating that the positive contributions of biodiversity to ecosystem functioning are increasingly compromised under saline conditions. When salinity deviates notably from typical environmental levels, the negative effects of biodiversity loss on ecosystem function become more pronounced.

Additionally, our analysis revealed a linear decline in the intercept of the diversity‐functioning relationship, which represents average community yield at intermediate levels of species richness, as salinity increased (*R* ^2^ = 0.97, *p* < 0.001; Figure 2B). This decline is likely attributable to osmotic stress that disrupts microbial resource‐use efficiency. Elevated salinity contributes to ion imbalances and cellular dehydration, resulting in reduced per‐capita productivity and suppressed asymptotic biomass, even under constant resource availability^33^. Therefore, high salinity conditions hinder microbial activity, dampening overall population viability and resultant biomass. These findings align with a growing body of evidence indicating that environmental conditions, such as salinity, can substantially modulate the effects of biodiversity on ecosystem function^27^, ^34^. For example, a previous study highlighted that temperature fluctuations could similarly alter the BEF relationship, with deviations from ambient conditions making the relationship more pronounced^24^. Furthermore, environmental complexity is believed to enhance the positive influence of species richness on ecosystem functioning through stronger complementarity effects, which include mechanisms such as resource partitioning and facilitated interactions among species when resources are plentiful^35^. Consequently, it is reasonable to conclude that salinity serves as a significant factor leading to shifts in the BEF relationship, emphasizing the importance of environmental context in shaping ecosystem dynamics.

Furthermore, our results underscore the necessity of higher species diversity to sustain ecosystem function under high salinity conditions (specifically when salinity exceeds 15% in this study). This aligns with previous research that identifies species diversity as a crucial factor in maintaining the stability of ecosystem functions, acting as a buffer against abiotic disturbances^19^, ^36^, ^37^. The insurance hypothesis posits that in diverse communities, the failure of certain species does not lead to the collapse of ecosystem functioning. Instead, these communities can provide a form of ecological insurance, ensuring that a variety of functions are maintained even when some species are compromised^38^, ^39^. This diversity allows different species to exploit available resources more efficiently, thereby enhancing overall ecosystem resilience in the face of unpredictable environmental conditions. Therefore, our results highlight the vital importance of protecting biodiversity as a primary strategy for sustainable ecosystem management. As global environmental changes, including rising salinity levels, pose significant challenges to ecosystems, safeguarding diverse microbial communities will be essential for maintaining ecosystem resilience and functionality. This approach can help mitigate the impacts of abiotic stressors and support the long‐term stability of ecosystem services critical to both human well‐being and ecological health^32^.

### Effects of salinity on the community compositions and metabolic function patterns

Our findings highlight the significant impact of salinity on microbial community composition within the highest‐richness consortium that was initially homogenized. We observed a clear progression in community composition from halosensitive genera, such as *Pseudomonas* and *Paenibacillus*, to halotolerant/halophilic genera, including *Halomonas* and *Marinococcus*, as salinity levels increased (Figure 3C). This shift is likely attributed to the varying levels of salt tolerance among different species, with more salt‐tolerant organisms progressively outcompeting those that are less tolerant at elevated salinities^10^. Salinity acts as an environmental filter that selectively excludes microbial species with similar ecological niches that cannot cope with increased salt concentrations. This observation aligns with previous research indicating that *Pseudomonas* and *Paenibacillus* are predominantly found in freshwater or low‐salinity ecosystems^40^, ^41^, ^42^. In contrast, genera such as *Halomonas* and *Marinococcus* are prevalent in marine or saline/hypersaline environments^43^, ^44^, ^45^, ^46^. The distinct differences in microbial community composition that we observed are consistent with existing literature, reinforcing the notion of unique microbial communities in freshwater versus saline or hypersaline lakes^47^, ^48^. These variations in community structure can be elucidated through the physiological constraints imposed by salinity, such as osmoregulation challenges, limited energy availability, and reduced water access that affect microbial survival and growth^49^, ^50^, ^51^. Consequently, the results of our study demonstrate that changes in salinity significantly influence the composition of microbial communities by altering species interactions, primarily driven by inherent differences in salt tolerance traits among microbial taxa. Overall, this emphasizes the crucial role of salinity in shaping microbial diversity and community dynamics, which has important implications for understanding ecosystem responses to changing environmental conditions.

The observations from our study on the highest‐richness microbial consortiums reveal notable patterns in enzyme activity and carbon source utilization across the five experimental salinity gradients, underscoring the significant impact of salinity on metabolic functions. Specifically, we found that microbial consortiums at low salinities exhibited higher levels of enzyme activity and greater capacity for carbon source utilization compared to those in high‐salinity environments (Figure 3). This aligns with previous research indicating that salinity can inhibit extracellular enzyme activity and profoundly affect essential metabolic pathways and functions^8^, ^52^, ^53^, ^54^. For example, our previous study indicated that *Gammaproteobacteria* from low‐salinity lakes demonstrated superior carbon utilization capacity (as measured by Average Well Color Development, AWCD) compared to their counterparts from high‐salinity lakes^55^. It is essential to note, however, that salinity does not have a uniform suppressive effect on metabolic activity. Instead, it appears to shift metabolic preferences within microbial communities. Under elevated salinity conditions (both middle and high), we observed a marked increase in the utilization of amines compared to the low salinity condition (Figure 3E). This shift likely represents an adaptive strategy for microbial communities to manage osmotic stress. The utilization of amines has been well‐documented as a metabolic strategy among halophiles, allowing them to maintain cellular homeostasis and support growth in extreme saline environments^56^, ^57^, ^58^. These findings highlight the complex and nuanced responses of microbial metabolic functions to variations in salinity. They reflect the adaptive resilience of microbial communities that can flexibly adjust their metabolic pathways in response to osmotic stress, thus ensuring their survival and functionality in fluctuating environmental conditions. In summary, our research illustrates that while increased salinity can limit some metabolic functions, microbial communities can also demonstrate remarkable adaptability through shifts in substrate utilization, emphasizing their resilience and metabolic flexibility in challenging environments.

### The contribution of selection and complementarity effects to the BEF relationship varied with salinity

The interplay between selection and complementarity effects serves as a fundamental basis for understanding how biodiversity enhances ecosystem biomass production and stability^59^. Selection effects manifest when interspecific interactions promote the predominance of particularly productive species, whereas complementarity effects stem from niche differentiation, such as resource partitioning, or beneficial interactions among different species^22^, ^60^. Existing research presents a divided perspective on the relative importance of these effects in microbial communities: some studies highlight the predominant role of selection effects in shaping microbial diversity and its consequent influence on ecosystem functions^28^, ^29^, while others propose that complementarity effects are the primary drivers of these functions^27^, ^30^. It is crucial to recognize that selection and complementarity effects are not mutually exclusive; rather, they frequently operate in concert to bolster productivity and ecosystem resilience^61^. Aligned with this comprehensive understanding, our results indicate that both selection and complementarity effects work synergistically to contribute to the NBEs observed in this study (as illustrated in Figure 4 and detailed in Table S2). By demonstrating the collaborative influence of these mechanisms, our findings highlight the complexity of BEF relationships, particularly within microbial communities. This underscores the importance of considering both types of effects when assessing ecosystem responses to biodiversity changes. Ultimately, the joint effects of selection and complementarity not only enrich our comprehension of microbial ecosystem functions but also provide insights into the management and conservation of biodiversity in various environmental contexts.

In addition, our findings indicate that salinity significantly influences the relative contributions of selection and complementarity effects to NBEs. Notably, across the salinity range from 0 to 15%, we observed an increase in selection effects as salinity increased, while complementarity effects correspondingly weakened. This observation supports our initial hypothesis and reflects trends seen in other ecological systems, where the balance between selection and complementarity can be affected by various factors, including temporal dynamics, resource availability, and interactions with neighboring species^62^, ^63^. For example, in intercropping systems, it has been noted that complementarity effects are more pronounced in the absence of nitrogen fertilization, while selection effects tend to dominate when nitrogen is added^23^. Similarly, in microbial communities facing thermal extremes, enhanced complementarity effects suggest that species interactions are crucial in mediating BEF relationships under temperature stress^24^. In contrast to these observations, our study highlights that increased salinity strengthens selection effects, likely due to the pronounced environmental filtering mechanisms imposed by saline conditions, which is evidenced by shifts in taxonomic composition over salinity gradients. The divergence in these mechanisms underscores the context‐dependent nature of biodiversity influences: while thermal stress appears to enhance species complementarity, osmotic stress fosters competitive dominance among species. Collectively, these results emphasize the critical necessity of accounting for prevailing environmental conditions when evaluating the roles of selection and complementarity effects in influencing ecosystem functions. Such an understanding can provide deeper insights into how ecosystems may respond to changing environmental parameters and inform strategies for biodiversity conservation and management in various contexts of ecological stress.

### Limitations of this study and recommendations for future studies

Our study acknowledges several limitations inherent in the artificial community construction methodology, which warrant careful consideration when interpreting the BEF relationships. First, the assembly of artificial communities was influenced by a priori selection of strains based on salt tolerance and their taxonomic affiliations. While this design ensures experimental tractability, the selective recruitment might bias the observed biodiversity effects, as natural communities often exhibit stochastic colonization and broader functional redundancy. The randomization process under which these communities were formed was not entirely unrestricted in this study, potentially introducing biases that affect the applicability of our results. Second, the simplified nature of the artificial communities utilized in our microcosm experiments does not adequately reflect the intricate composition and dynamics found in natural ecosystems. In real‐world settings, microbial communities exhibit greater diversity and are subject to more frequent and varied interactions, which can significantly impact their functions. Moreover, the salinity ranges examined in our study did not encompass saturation levels, and we concentrated exclusively on salinity as a driving environmental factor. In nature, salinity can reach saturation, and it often interacts with other critical factors, such as temperature, nutrient availability, and pH, creating a complex web of influences that shape microbial community structure and ecosystem function. To address these limitations, future research should aim to validate our findings in natural environments where microbial communities demonstrate higher complexity and more dynamic environmental conditions. This validation is essential for confirming the relevance of our laboratory observations to real‐world scenarios. Additionally, we suggest that future studies investigate the possibility of a tipping point concerning salinity effects on BEF relationships. Leveraging predictive modeling approaches could aid in identifying critical thresholds for salinity and elucidating their ecological implications, providing deeper insights into how biodiversity dynamics might shift under varying environmental stressors. Such research would enhance our understanding of the resilience of ecosystems in the face of changing salinity and other environmental factors, ultimately contributing to more effective ecosystem management and conservation strategies.

In summary, our study reveals that salinity plays a systematic role in reshaping BEF relationships. Specifically, at low salinity levels, the effects on biodiversity and ecosystem functioning are minimal; however, as salinity increases, we observed significant declines in functional performance. Saltier communities generally supported lower levels of asymptotic biomass, indicating a reduction in ecosystem productivity under high salinity conditions. Importantly, while biodiversity enhancement under high salinity levels improved ecosystem functioning, it also partially mitigated the functional declines induced by salinity. Our results demonstrated that salinization not only altered microbial community compositions but also affected the metabolic patterns of the most diverse microbial consortiums. As salinity levels rose, the contribution of selection effects to the overall NBE gradually increased, eventually reaching a saturation point (e.g., around 15% salinity in this study). Concurrently, the contribution of complementarity effects diminished. When salinity exceeded this critical threshold (e.g., beyond 15%), we observed that only interspecific complementarity effects emerged, indicating that microbial communities with high diversity tended to exhibit functional redundancy. This finding underscored the potential consequences of biodiversity loss in high salinity environments, highlighting that maintaining high species diversity is essential for sustaining ecological functions as salinity increases.

## MATERIALS AND METHODS

### Field site and sampling

In May 2017, a sampling cruise was conducted in northern Qinghai Province, China. During the sampling campaign, six lakes with different salinity were selected for sampling: Erhai Lake (EHL), Qinghai Lake (QHL), Tuosu Lake (TSL), Gaihai Lake (GHL), Xiaochaidan Lake (XCDL), and Chaka Lake (CKL) (Figure S2). The salinity of the sampled lakes ranged from 0.08% to 34.19%. A detailed description of the geochemistry of these lakes can be found in our previous studies^64^, ^65^. Surface sediments (approximately 1–5 cm deep) were collected at the field site using a grab bucket collection sampler XDB0201 (New Landmark). Sediment samples from each lake were collected into sterile Falcon tubes for microbial cultivation. All sediment samples were transported to the laboratory under cold and dark conditions, where they were stored in refrigerators at a temperature of 4°C until microbial cultivation.

### Isolation, cultivation, and identification of bacterial taxa from different saline lakes

Bacterial strains were isolated using the standard dilution plating method. Briefly, approximately 5 g of homogenized sediment sample was suspended in an Erlenmeyer flask containing 45 ml of sterile water (the salinity was the same as the lake salinity) and shaken on a rotary shaker at 28°C at 180 rpm for 1 h. The resulting suspensions were serially diluted to a concentration of 10^−4^, and each of the dilutions (200 μl) was plated into eight types of enrichment media^65^. The detailed composition and formulation of the enrichment media were provided in Table S5. All plates were incubated in the dark at 28°C for 2 weeks. Differentiated colonies were then picked from these enrichment media and purified on their respective medium plates. Well‐isolated colonies were preserved as glycerol suspensions (20%, v/v) at −80°C. Isolates were assigned a taxonomy by colony PCR using a universal primer set (27F and 1492R) for the 16S rRNA gene. PCR amplification of the 16S rRNA gene and sequence processing were followed by the previously described procedures^66^. A total of 646 strains were obtained, which were assigned to 4 bacterial phyla, 7 classes, 18 orders, 39 families, 89 genera, and 210 species^65^. Preliminary salt tolerance screening was conducted on these culturable strains, and finally 40 representative isolates from the six lakes were selected for the subsequent experiments (Figure S3 and Table S6). Strains were selected based on their salt tolerance and phylogenetic diversity to ensure a representative microbial community. Artificial communities were then constructed to simulate varying richness levels, allowing us to examine the impact of salinity on BEF relationships. The phylogenetic tree was constructed with neighbor‐joining algorithms^67^ using the MEGA version 7.0 software package. For visualization enhancement and a graphical representation, the tree topology was further processed and annotated using the “ggtree” package^68^ in the R environment. The phylogenetic tree constructed based on 16S rRNA gene sequences and taxonomy identification revealed that the 40 selected isolates belonged to 4 phyla, 10 orders, 16 families, and 32 genera (Figure S3 and Table S6). These strains were distributed among *Actinobacteria* (6 strains, accounting for 15.0%), *Bacteroidetes* (5 strains, 12.5%), *Firmicutes* (19 strains, 47.5%), and *Proteobacteria* (10 strains, 25.0%).

### Salinity tolerance of the selected bacterial strains

For bacterial salt tolerance tests, all 40 isolates were individually cultured in Reasoner 2A (R2A) agar medium for 72 h at their optimal salt content. Subsequently, observable colonies were transferred to the R2A liquid medium and prepared as suspensions. Biomass in suspension was estimated by measuring optical density (OD) at 600 nm. Diluted suspensions (0.1 OD_600_) were used as initial inoculum. To characterize the salinity tolerance capacities, each bacterial strain (1 ml) was cultured in three flasks with 100 ml of liquid R2A medium supplemented with different concentrations of NaCl: 0, 2.5%, 5%, 7.5%, 10%, 12.5%, 15%, 20%, 25%, and 30% (w/v) (Figure 1A). The flasks were incubated at a temperature of 28°C with stirring at 180 rpm for 120 h. To quantify bacterial biomass along the salinity gradient, the absorbance of each sample was measured at 600 nm using a spectrophotometer (UV1750; Shimadzu). The standard deviation is based on three independent cultivations. The salt tolerance screening demonstrated that all 40 bacterial strains exhibited robust growth within their respective optimal salinity ranges (Figure S4). Based on these physiological tolerances and phylogenetic diversity, we systematically assembled artificial microbial communities with increasing diversity, ensuring their viability across the experimental salinity gradients.

### Experimental design of BEF experiment

The experimental design was inspired by a previous study^24^, with modifications. Specifically, four richness levels of artificial communities were established: 5 strains, 10 strains, 20 strains, and 40 strains, corresponding to Group A, B, C, and D, respectively (Figure 1B). The artificial communities were constructed based on salinity tolerance traits and phylogenetic diversity, with Group A being assembled through random selection of 5 strains from a pool of 40 representative isolates (capable of growth under different salinity treatments). Similarly, Group B was formed by randomly selecting 10 strains from the same pool, with the same approach being applied to Group C and D. The experimental design was developed to account for combinatorial possibilities, with replicates being established as follows: Group A was represented by 4 distinct replicates (designated as A1, A2, A3, and A4; all replicates were generated independently), Group B by 8 distinct replicates, and Group C by 16 distinct replicates. Note that these replicates were randomly selected from nonoverlapping combinatorial pools, with the experimental design emphasizing ecological relevance rather than complete randomization. For Group D, where only one unique combination was possible, 3 replicates were maintained to ensure experimental robustness. Detailed information regarding species composition is shown in Table S7. These randomly assembled artificial communities were then grown at each level of species richness at five different salinities: 0.9%, 3.5%, 7%, 15%, and 20% (the salinities at which all assembled communities could grow). Stock cultures of all 40 isolates were grown to the stationary phase at each of the experimental salinity treatments and diluted back to a common biomass density for all isolates and salinity treatments. The assembled artificial communities were inoculated from these stock cultures to a standardized target biomass and microcosm volume (40 ml) across all salinity treatments. For example, at a species richness of 5 (Group A), 8 ml of each species was added, while at a species richness of 10 (Group B), 4 ml of each was added (see Table S6 for more detailed information). Then, a 40 ml volume of microcosm was suspended in flasks containing 500 ml sterile liquid R2A medium and maintained in an orbital shaker (28°C, 50 rpm). After 120 h of incubation, the homogenized suspensions of the assembled communities were then used to assess ecological function (i.e., biomass yield) and metabolic functions (i.e., enzyme activity and carbon utilization).

The OD_600_ of the assembled artificial communities across five experimental salinity gradients (0.9%, 3.5%, 7%, 15%, and 20%) was measured after 120 h (when the stationary phase was reached) with a spectrophotometer. Ecological function was quantified as the asymptotic biomass (i.e., yield) of the artificial communities in the stationary phase. To quantify the strength of the BEF relationship across salinity gradients, we applied a power function to model the relationship between species richness and ecosystem function under varying salinities, and quantified the effects of salinity on the exponent and intercept, using a linear mixed‐effects model. The experiment structure was well defined by a power function^24^:

(1) $$\log_{10}Y\left(S\right)=b\left(\log_{10}S-\log_{10}S_{c}\right)+\log_{10}Y\left(S_{c}\right)$$

Here, $\log_{10}Y(S)$ represents the natural logarithm of ecosystem functioning (quantified as biomass yield, *Y*, during the stationary phase of community growth) at a given species richness (*S*), *S_c_* denotes mean‐centered species richness, and *b* is the exponent that characterizes the shape of the diversity‐functioning relationship. By centering the species richness variable, we set the intercept, log_10_ *Y*(*S*_*c*_), to represent the yield at the average level of species richness. The exponent *b* provides critical insights: when *b* < 1, ecosystem functioning decelerates with increasing species richness, indicating functional redundancy; when *b* = 1, functioning increases linearly with richness, suggesting no functional redundancy and a positive contribution of each species to ecosystem functioning. To quantify the effects of salinization on this relationship, we fitted the equation to experimental data using a linear mixed‐effects model via the “lmer” function in the “lme4” package of R software. Salinity was included as a fixed categorical factor to evaluate how *b* and $\log_{10}Y\left(S_{c}\right)$ varied across different salinity treatments.

To further elucidate the functional consequences of salinity stress and biodiversity loss, we systematically quantified metabolic responses through extracellular enzyme profiling and carbon substrate utilization assays. Extracellular enzyme activities were characterized using API ZYM strips (BioMerieux), a standardized semi‐quantitative method for assessing 19 hydrolytic enzymes. Community‐level metabolic profiles were evaluated through Biolog EcoPlate analyses, which measure the utilization kinetics of 31 ecologically relevant carbon substrates. Comprehensive methodological details and analytical protocols for both functional assessments are provided in the Supplementary Information.

### Effects of salinity on community composition

At the end of the BEF experiment, we examined the community compositions in Group D, which represented treatments with the highest diversity (richness = 40) and were cultivated at five different salinities (0.9%, 3.5%, 7%, 15%, and 20%). Detailed procedures for DNA extraction, PCR preparation, sequence processing, and associated statistical analyses were provided in the Supplementary Information.

### Quantifying the contribution of selection and complementarity effects to BEF interactions

To statistically partition the NBE into its complementarity effect and selection effect components (Figure 1C), we followed a previous additive partitioning method^22^:

(2) $$\Delta Y=Y_{O}-Y_{E}=\sum_{i}RY_{O,i}M_{i}-\sum_{i}RY_{E,i}M_{i}=\sum_{i}\Delta RY_{i}M_{i}=N\bar{\Delta \mathrm{RY}}\bar{M}+N\text{cov}(\Delta \mathrm{RY},M)$$

In this equation, $Y_{O}$ is the observed total yield of the mixture, $Y_{E}$ is the expected total yield of the mixture; therefore, ΔY = NBE means a deviation from the expected total yield in the mixture. Where $M_{i}$ is the yield of species *i* in monoculture, $RY_{O,i}$ and $RY_{E,i}$ are the observed relative yield of species *i* in the mixture and the expected relative yield of species *i* in the mixture (simply the added proportion in the mixture), respectively, and $\Delta RY_{i}$ is the deviation from the expected relative yield of species *i* in the mixture. $N\bar{\Delta \mathrm{RY}}\bar{M}$ is the complementarity effect, where *N* is the number of species in the mixture, $\bar{\Delta \mathrm{RY}}$ is the average change in the relative yield for all species in the mixture, and $\overset{®}{M}$ is the average monoculture yield; Ncov(ΔRY,M) is the selection effect, calculated as the covariance between the monoculture yield of the species and their change in relative yield in the mixture^69^. Using information on the relative abundance of genera retrieved from the highest diversity treatments cultured at five different salinities (0.9%, 3.5%, 7%, 15%, and 20%), the NBE was quantified as the difference between the observed yields of the mixture and the expected yield of the mixture (weighted average yield of the monocultures)^24^.

Linear regression analyses were performed using the “vegan” and “ggplot2” packages to assess the correlation between NBE/selection effect/complementarity effect and salinity. ANOVA was conducted using the “aov” function to test for significant differences between different salinity treatments for each mechanism (NBE, selection effect, complementarity effect), with the significance level set at *p* < 0.05. In addition, Tukey's HSD tests were employed via the “TukeyHSD” function to identify significant (*p* < 0.05) differences between all pairwise salinity levels.

## AUTHOR CONTRIBUTIONS

**Jianrong Huang**: Data curation; formal analysis; investigation; methodology; software; validation; visualization; writing—original draft. **Mingxian Han**: Data curation; formal analysis; methodology; visualization; writing—original draft. **Jian Yang**: Conceptualization; data curation; formal analysis; methodology; supervision. **Yi Wang**: Formal analysis; funding acquisition; methodology; supervision. **Hongchen Jiang**: Conceptualization; funding acquisition; investigation; project administration; supervision; writing—review and editing.

## ETHICS STATEMENT

No animals or humans were involved in this study.

## CONFLICT OF INTERESTS

The authors declare no conflict of interests.

## Supporting information

Additional Supporting Information for this article can be found online at doi.org/.

## Data Availability

The 16S rRNA gene sequences of 40 strains were assigned accession numbers OP804654–OP804692 and OP805340 in the NCBI GenBank database. All the raw sequences obtained from this study have been deposited in the NCBI Sequence Read Archive (SRA) database under project PRJNA1097298 with accession numbers SRR28579863–SRR28579877. All data needed to evaluate the conclusions in the article are present in the article and/or the Supplementary Information.
