# Supplementary material for: Salinization influences the biodiversity‐ecosystem functioning relationship more strongly at high salinity
Source: mLife. 2025 Nov 18;4(6):683–96. doi: 10.1002/mlf2.70034 (PMC12754629; doi:10.1002/mlf2.70034)
Supplement: Supplementary file 1 — Additional Supporting Information for this article can be found online at doi.org/. [file MLF2-4-683-s001.docx]

Supplementary Information for

**Salinization influences the biodiversity-ecosystem functioning relationship more strongly at high salinity**

Jianrong Huang^1,2^, Mingxian Han^1^, Jian Yang^1^*, Yi Wang^3^, Hongchen Jiang^1,2^*

^1^School of Life Sciences, Henan University, Kaifeng 475001, China.

^2^Qinghai Provincial Key Laboratory of Geology and Environment of Salt Lakes, Qinghai Institute of Salt Lakes, Chinese Academy of Sciences, Xining 810008, China.

^3^School of Mathematics and Physics, China University of Geosciences, Wuhan 430074, China.

***Correspondence:** Hongchen Jiang, Hongchen_jiang@126.com; Jian Yang, yangjiancug@126.com

**This PDF file includes:**

Supplementary Text

Figs. S1 to S6

Tables S1 to S7

**Supplementary Text**

**Influence of salinity and species loss on metabolic functions (****enzyme activity and carbon utilization)**

Each API ZYM strip consists of three phosphatases, three esterases, three aminopeptidases, two proteases, and eight glycosyl hydrolases [[1](#_ENREF_1)]. An aliquot of the homogenized suspensions (135 μl) was dispensed into microcupules containing various dehydrated chromogenic substrates for specific enzymes. To create a humid atmosphere, sterile distilled water was added to the plastic outer cover. The API ZYM strips were then incubated at 28°C for 24 h. After incubation, commercial reagents ZYM A and ZYM B (one drop each; bioMérieux) were added to each microcupule as recommended by the manufacturer. Color reactions were observed after 5 min at room temperature. Each microcupule was scored as positive (color development) or negative (no color development). Positive color development was quantified using a numerical value ranging from 1 to 5 based on the color chart provided by the manufacturer. The results were then classified into low-intensity (1), medium-intensity (2–3), and high-intensity (4–5) responses, according to the protocols of previous studies [[1-3](#_ENREF_1)]. The color intensity measurements of the enzymatic reactions within each microcupule were conducted by three independent observers. While it is crucial to note that the ZYM results may exhibit limitations in precision, they nonetheless provide invaluable insights into the fluctuations of enzyme activity across artificially constructed communities exposed to varying levels of salinity and species richness.

The Biolog EcoPlate™ (Biolog, Inc., Hayward, CA, USA) is a tool for detecting the metabolic functions of microbial populations. It consists of a 96-well plate containing 31 different carbon compounds (categorized into 6 biochemical categories) and a control, allowing a triplicate experiment on one plate. Each well was supplemented with tetrazolium violet dye, which turns purple when the microbial communities in the sample utilize the carbon source [[4](#_ENREF_4), [5](#_ENREF_5)]. The absorbance of each well directly indicated the capability of carbon source utilization in microbial communities and was measured using average well-color development (AWCD) [[6](#_ENREF_6), [7](#_ENREF_7)]. For this test, suspensions of assembled communities obtained at the end of the biodiversity ecosystem functioning experiment were diluted with a saline solution (0.9%) and homogenized on a horizontal shaker for 10 min. The resulting suspension (125 μl) was then pipetted into each microtiter well. The Biolog EcoPlates with lids were then incubated at 28°C under dark and humid conditions, while absorbance measurements were taken at every 24-hour interval over a period of 120 hours using an Emax precision microplate reader equipped with an autosampler (Emax precision microplate reader, Molecular Devices, Sunnyvale, USA). The AWCD value was calculated using the equation (1) [[8](#_ENREF_8)]:

AWCD=$\sum_{i=1}^{n} (Ci-R)/n$ (1)

where *Ci* is the absorbance value of each reaction well at 590 nm, *R* is the absorbance value of the control well, and *n* is the number of substrates, in this case *n* = 31. To analyze the AWCD of all carbon sources the substrates were divided into six categories representing different substrate guilds: carbohydrates, amino acids, esters, sterols, amines and carboxylic acids [[9](#_ENREF_9)].

All statistical analyses were conducted employing R software (version 4.3.2, <http://cran.r-project.org/>) unless otherwise indicated. The “ComplexHeatmap” package was utilized to create heatmaps that visualize the intensity of extracellular enzyme activities (Fig. S5) and the capacity for carbon source utilization (Fig. S6) in the artificially assembled communities cultured across five distinct salinity gradients (0.9%, 3.5%, 7%, 15%, and 20%). Linear regression analyses, performed utilizing the “basicTrendline” package, elucidated the relationships between metabolic functions (enzyme activities and carbon utilization) and salinity, particularly as species richness increased. Moreover, the “ggplot2” package was employed to generate histograms that depict the metabolic intensity profiles of five enzyme categories and six patterns of carbon source utilization within the highest-diversity treatments (Group D, richness = 40) cultivated at the aforementioned salinities.

**Effects of salinity on community composition**

For microbial community analyses, approximately 100 ml of suspension from the highest diversity treatments was aseptically filtered through 0.2 μm nuclepore membrane filters (Whatman, Maidstone, UK) into a sterilized 1.5 ml tube. The resulting biomass-containing filters were stored at −80°C in the laboratory until further processing. DNA extraction from biomass-containing filters was performed using the Fast DNA SPIN Kit for Soil (MP Biomedical, Santa Ana, USA) according to the manufacturer's instructions. The V4 region of the bacterial 16S rRNA gene was amplified with the uniquely tagged primer pair 515F (5’-GTGYCAGCMGCCGCGGTAA-3’) and 806R (5’-GGACTACNVGGGTWTCTAAT-3’) [[10](#_ENREF_10)]. PCR amplification, sequencing and data processing followed descriptions in our previous studies [[11](#_ENREF_11), [12](#_ENREF_12)]. Amplicon sequencing was performed using an Illumina-Miseq 2500 platform (paired-ends sequencing of 2 × 250 bp).

Nonmetric multidimensional scaling (NMDS) analysis at the ASV (Amplicon Sequence Variants) level was conducted to determine whether community composition at the end of the biodiversity ecosystem functioning experiment was significantly different among different salinity treatments (0.9%, 3.5%, 7%, 15% and 20%). The R package “vegan” was used for NMDS analyses, using Bray-Curtis dissimilarity to calculate community dissimilarity. Linear regression using the “basicTrendline” package was employed to assess the correlation between species scores extracted from NMDS1 and salinity. Permutational multivariate analysis of variance (PERMANOVA) was performed to test the effects of salinity on community composition at the end of the experiment (permutations = 999, method = ‘bray’), using the “adonis” function in the “vegan” package [[13](#_ENREF_13)]. For each pair of salinity treatments, separate PERMANOVA analyses based on the NMDS results from this study (e.g., low salinity-medium salinity, low salinity-high salinity, medium salinity-high salinity) were performed to determine which pairwise treatment contrasts differed significantly.

**References**

1. Patel D, Gismondi R, Alsaffar A, Tiquia-Arashiro SM. Applicability of API ZYM to capture seasonal and spatial variabilities in lake and river sediments. Environ Technol. 2019; **40:**3227-3239.

2. Tiquia SM, Wan HC, Tam NFY. Microbial population dynamics and enzyme activities during composting. Compost Sci Util. 2002; **10:**150-161.

3. Martínez D, Molina MJ, Sánchez J, Moscatelli MC, Marinari S. API ZYM assay to evaluate enzyme fingerprinting and microbial functional diversity in relation to soil processes. Biol Fertility Soils. 2016; **52:**77-89.

4. Oest A, Alsaffar A, Fenner M, Azzopardi D, Tiquia-Arashiro SM. Patterns of change in metabolic capabilities of sediment microbial communities in river and lake ecosystems. Int J Microbiol. 2018; **2018:**6234931.

5. Gryta A, Frąc M, Oszust K. The application of the Biolog EcoPlate approach in ecotoxicological evaluation of dairy sewage sludge. Appl Biochem Biotechnol. 2014; **174:**1434-1443.

6. Stefanowicz A. The Biolog plates technique as a tool in ecological studies of microbial communities. Pol J Environ Stud. 2006; **15:**669-676.

7. Garland JL, Mills AL. Classification and characterization of heterotrophic microbial communities on the basis of patterns of community-level sole-carbon-source utilization. Appl Environ Microbiol. 1991; **57:**2351-2359.

8. Ge Z, Du H, Gao Y, Qiu W. Analysis on metabolic functions of stored rice microbial communities by BIOLOG ECO microplates. Front Microbiol. 2018; **9:**1375.

9. Miao L, Guo S, Liu Z, Liu S, You G, Qu H et al. Effects of nanoplastics on freshwater biofilm microbial metabolic functions as determined by BIOLOG ECO microplates. Int J Env Res Public Health. 2019; **16:**4639.

10. Walters W, Hyde ER, Berg-Lyons D, Ackermann G, Humphrey G, Parada A et al. Improved bacterial 16S rRNA gene (V4 and V4-5) and fungal internal transcribed spacer marker gene primers for microbial community surveys. mSystems. 2015; **1:**e00009-15.

11. Yang J, Jiang H, Liu W, Huang L, Huang J, Wang B et al. Potential utilization of terrestrially derived dissolved organic matter by aquatic microbial communities in saline lakes. ISME J. 2020; **14:**2313-2324.

12. Huang J, Yang J, Jiang H, Wu G, Xie Z, Dong H. Surviving onshore soil microbial communities differ among the Qing-Tibetan lakes with different salinity. FEMS Microbiol Ecol. 2019; **95:**fiz156.

13. Oksanen J, Blanchet FG, Kindt R, Legendre P, Minchin P, O’Hara R et al. Vegan community ecology package: ordination methods, diversity analysis and other functions for community and vegetation ecologists. R package, Version. 2015**:**2-3.


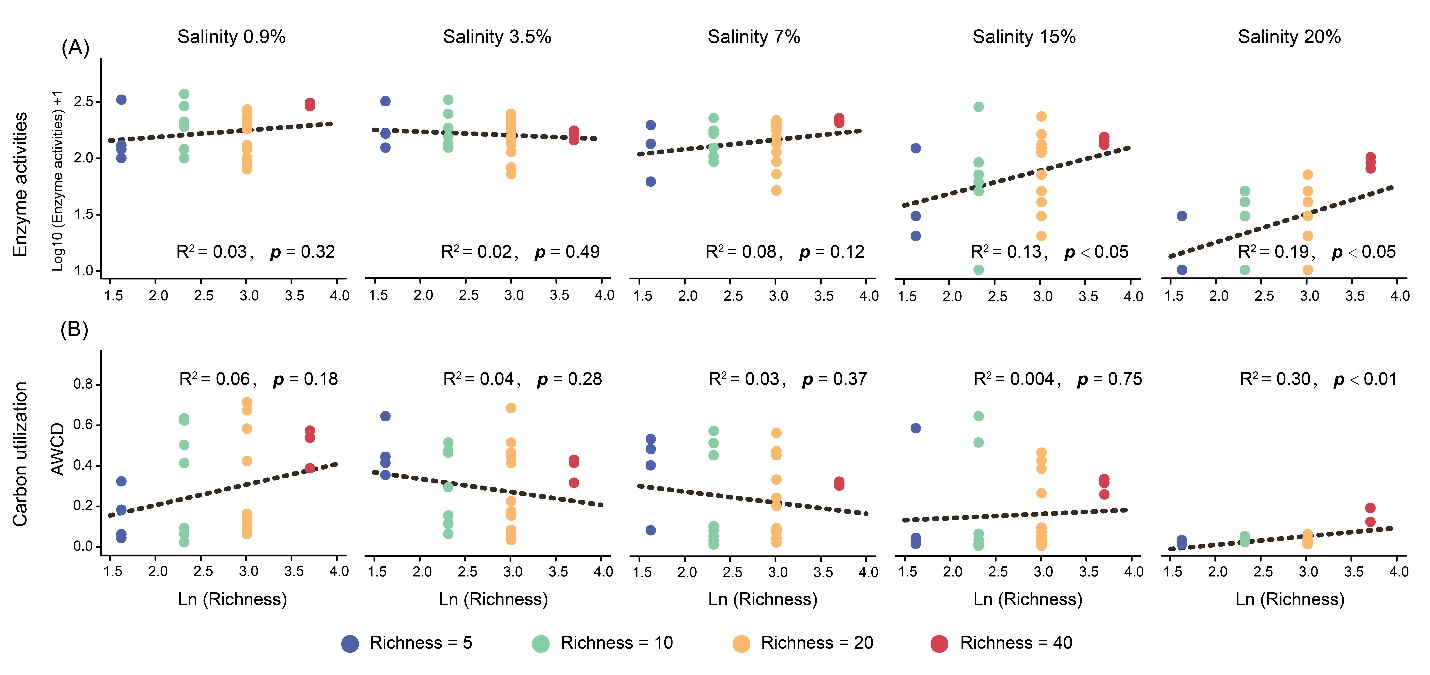


**Fig. S1.** Linear regression analysis showing the effects of salinity and species richness on metabolic functions. Metabolic functions were quantified based on enzyme activity (A) and carbon utilization (B). The different colors represent the level of species richness (Sp.). The number of points at each salinity represents the distinct replicates.


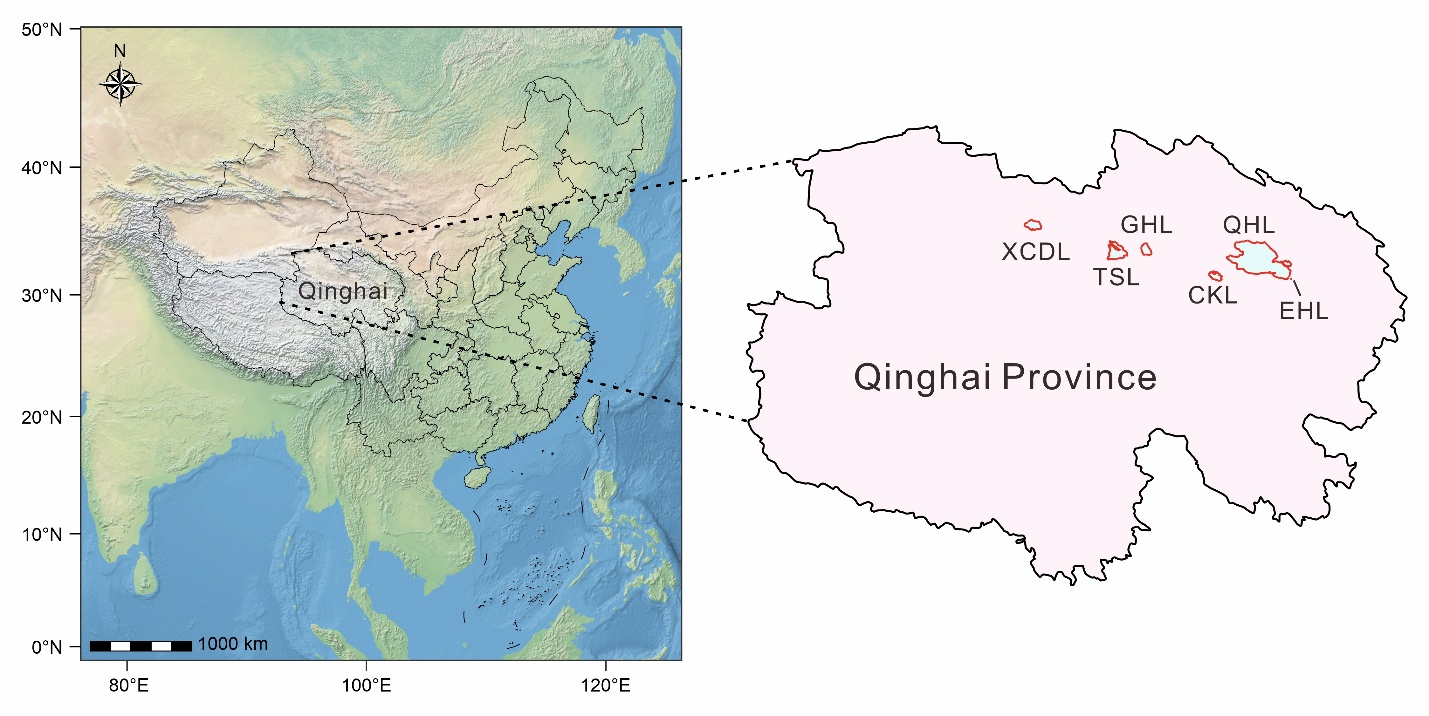


Fig. S2. A map showing the geographical locations of the six studied lakes in northern Qinghai Province, China. EHL: Erhai Lake, QHL: Qinghai Lake, TSL: Tuosu Lake, GHL: Gahai Lake, XCDL: Xiaochaidan Lake, CKL: Chaka Lake.


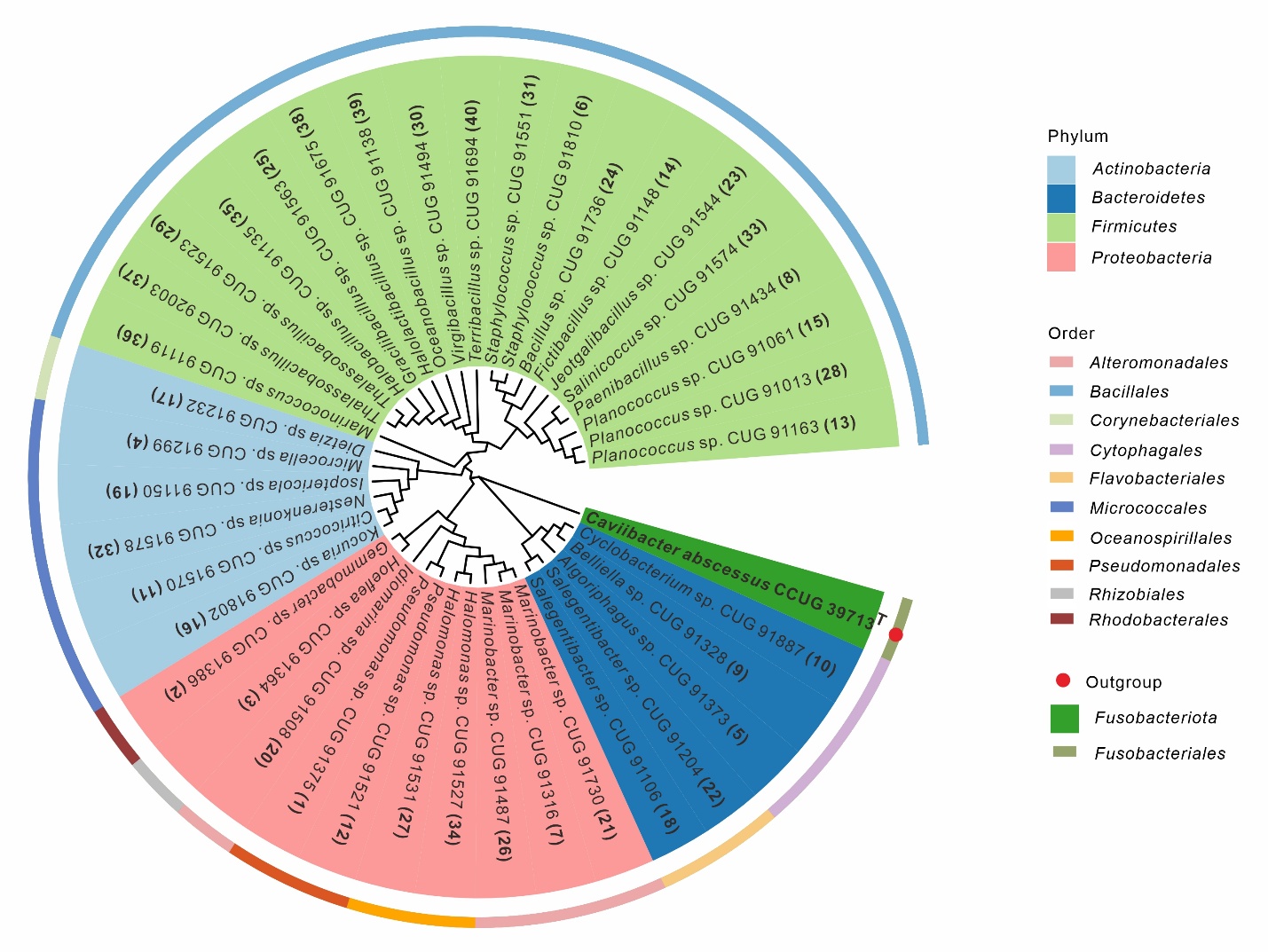


Fig. S3. Phylogenetic tree based on 16S rRNA gene sequences showing the phylogeny of 40 different strains isolated from lake sediments. Taxonomic details and associated information for each strain are provided in Table S6. *Caviibacter abscessus* CCUG 39713^T^ was used as the outgroup. Leaves are color-coded according to their respective phyla/orders.


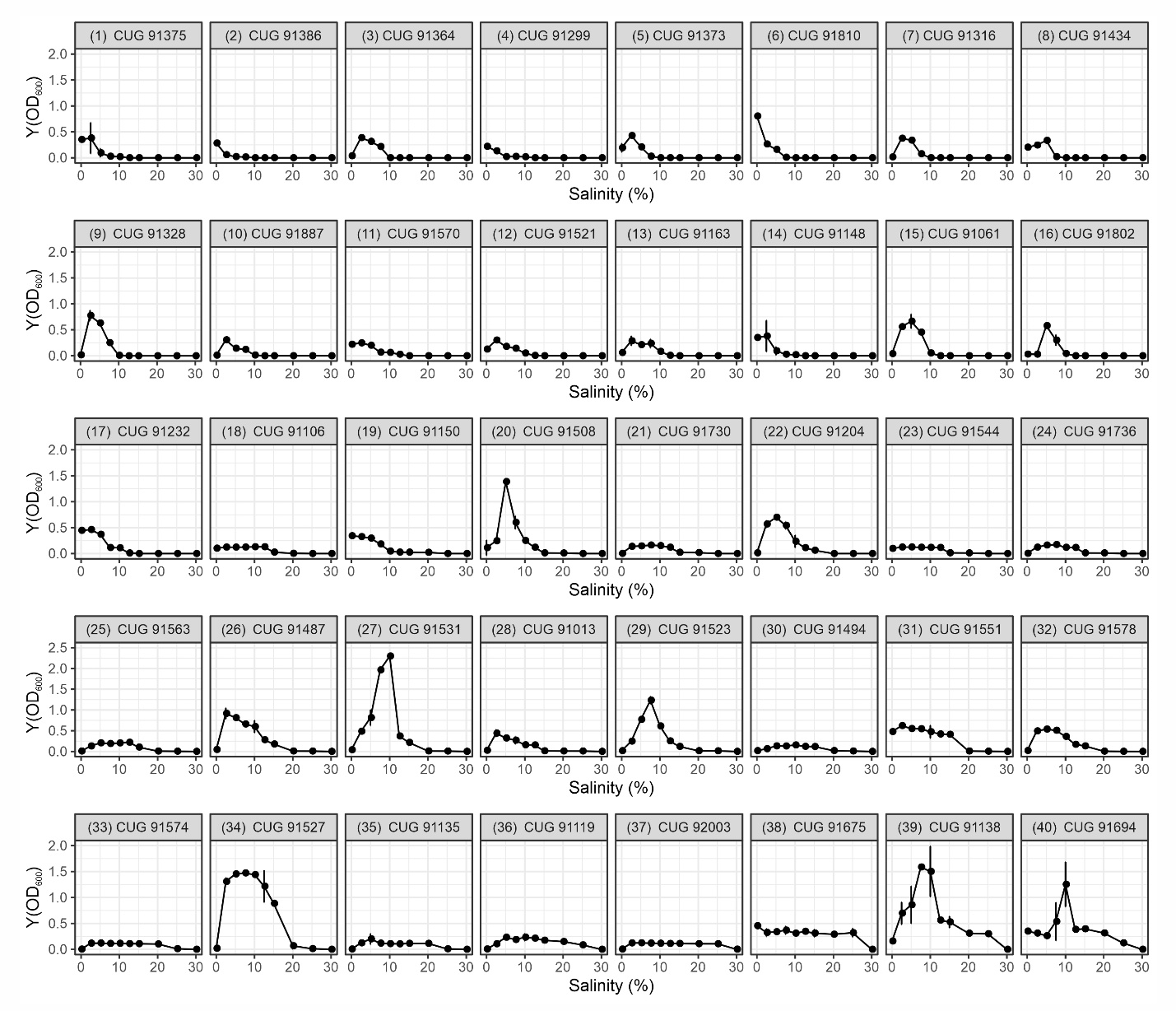


Fig. S4. Salt tolerance curves of the 40 isolates recorded after 5 days of incubation. The Y-axis represents the average optical density (OD) of each strain measured at 600 nm. The X-axis represents the growth salinity (%). All data points are mean of three replicates; Error bars represent standard deviations calculated from triplicates. The number in parentheses is the identity of each isolated taxa (see Table S6).


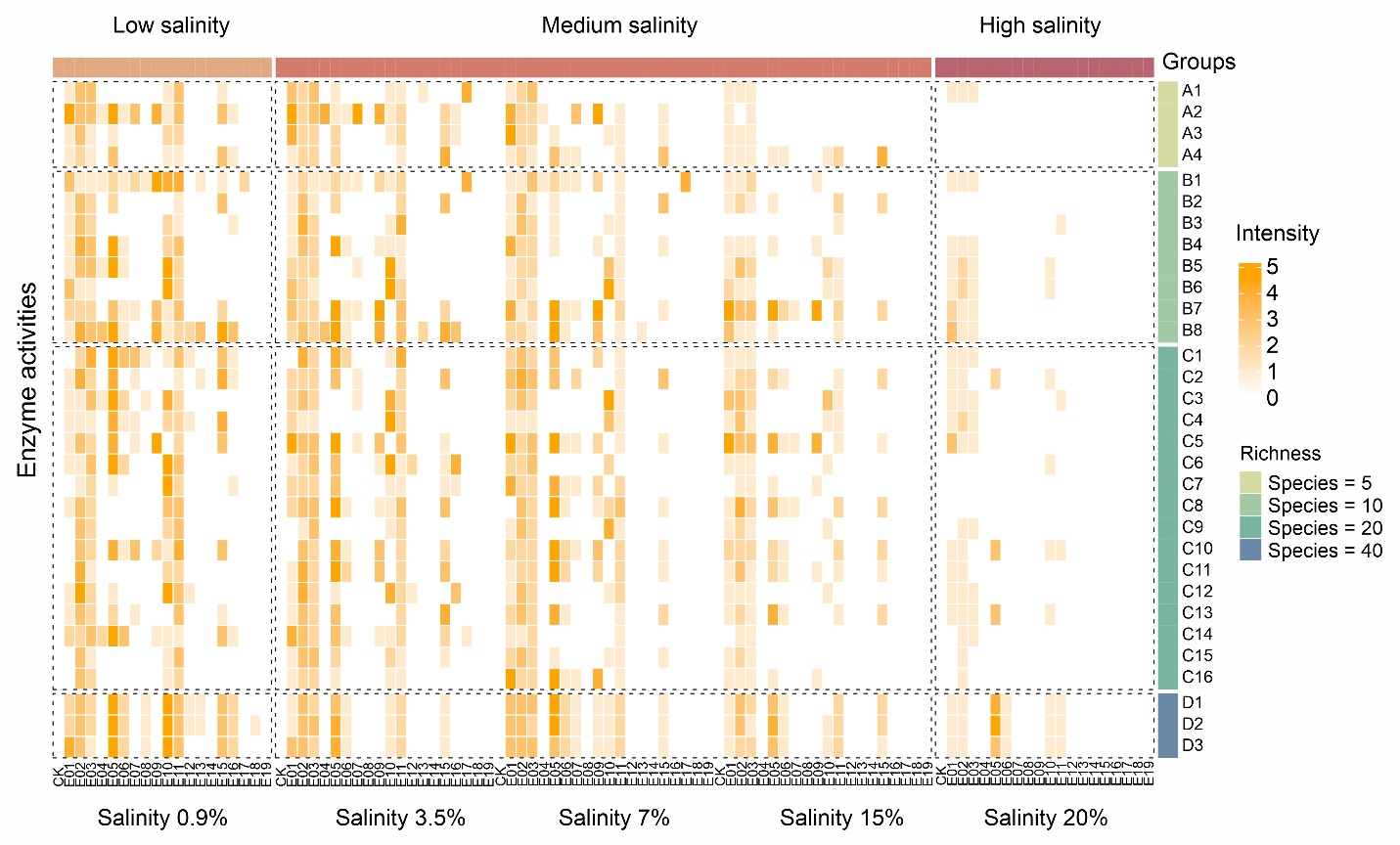


Fig. S5. Profiles of the 19 extracellular enzyme activities assessed by the API ZYM system from the assembled artificial communities cultivated at five different salinities (0.9%, 3.5%, 7%, 15% and 20%). The shading in the boxes indicates the intensity of the enzyme activities. The positive microcupules were assigned a numerical value of 1–5 according to the color chart provided by the manufacturer. Values are as follows: White: not detected; light orange: low intensity reactions (scores 1); medium orange: medium intensity reactions (scores 2–3); dark orange: high intensity reactions (scores 5).


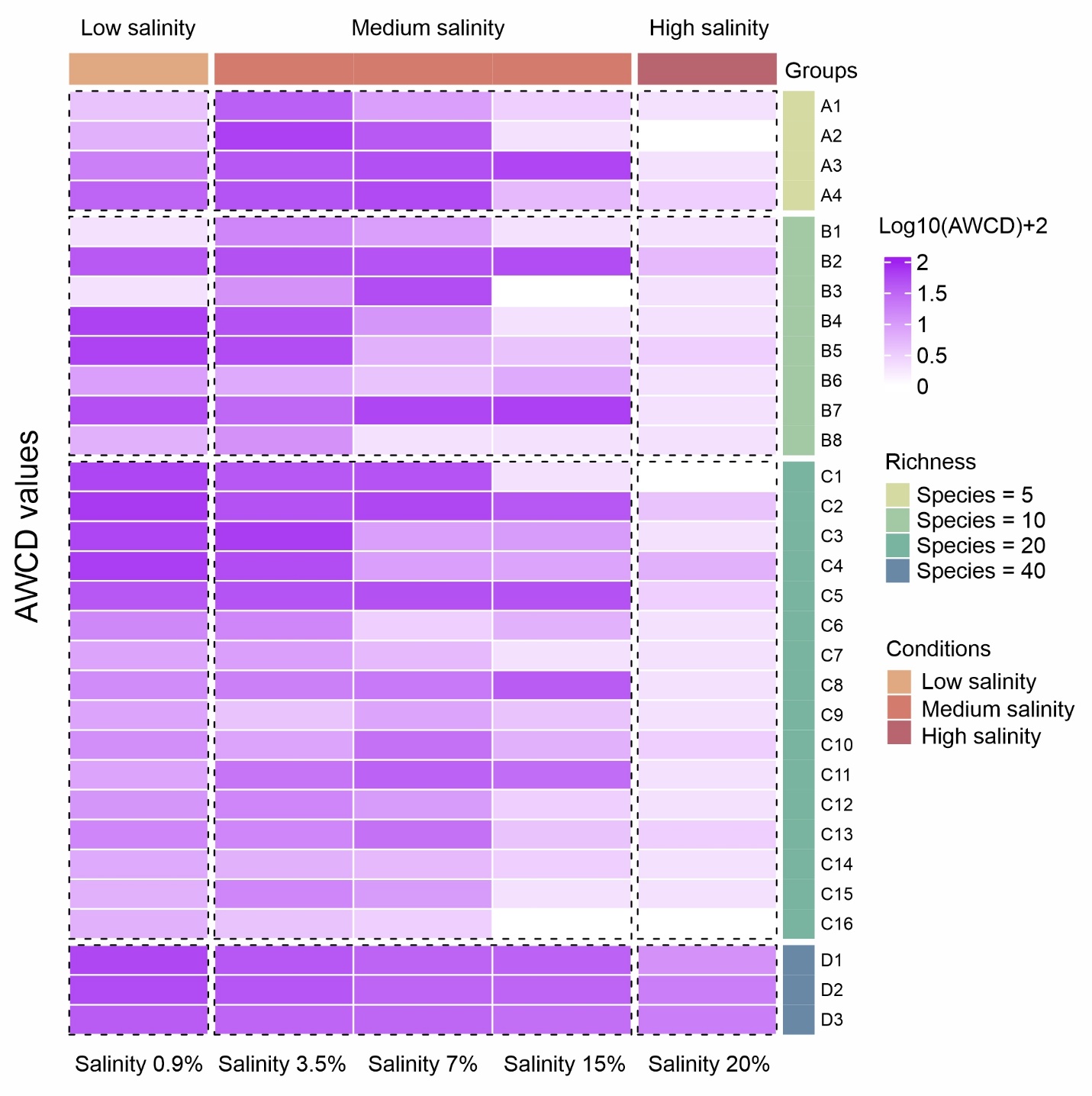


Fig. S6. The average well color development (AWCD) of all carbon sources assessed with the BIOLOG ECO microplate system from the assembled artificial communities cultivated at five different salinities (0.9%, 3.5%, 7%, 15% and 20%). Light purple indicates low use of carbon source by the assembled artificial communities, and darker shades of purple indicate higher use.

Table S1. Permutational multivariate analysis of variance (PERMANOVA) test of differences in microbial community composition at different culture salinity based on Bray-Curtis distance measures (Permutation: 999).

| Group | R^2^ | *p* | Significance |
| --- | --- | --- | --- |
| All groups | 0.92 | 0.001 | *** |
| Low salinity / Medium salinity | 0.86 | 0.006 | ** |
| Low salinity / High salinity | 0.99 | 0.1 | / |
| Medium salinity / High salinity | 0.81 | 0.006 | ** |

Note: *** Correlation is significant at *p* < 0.001 level. * Significant at *p* < 0.01 level.

Table S2. Partitioning the effects of salinization on ecosystem function (biomass density) into selection and complementarity effects at the local scale.

| Salinity treatments | local net biodiversity effect (NBE) | local selection effect (SE) | local complementarity effect (CE) |
| --- | --- | --- | --- |
| 0.9% | 2.28±0.01 | -0.08±0.20 | 2.36±0.21 |
| 3.5% | 2.08±0.04 | 0.92±0.03 | 1.16±0.01 |
| 7% | 2.34±0.01 | 0.74±0.04 | 1.60±0.04 |
| 15% | 2.41±0.03 | 1.66±0.14 | 0.73±0.11 |
| 20% | 0.24±0.01 | -0.04+0.00 | 0.28±0.02 |

Table S3. Results from the analysis of variance (ANOVA) on the local scale biodiversity partitions.

| Parameter | Net biodiversity effect | | | | |  | Selection effect | | | | |  | Complementarity effect | | | | |
| --- | --- | --- | --- | --- | --- | --- | --- | --- | --- | --- | --- | --- | --- | --- | --- | --- | --- |
|  | Df | Sum of Sqs | Mean Sqs | F | *p* |  | Df | Sum of Sqs | Mean Sqs | F | *p* |  | Df | Sum of Sqs | Mean Sqs | F | *p* |
| Salinity | 4 | 10.19 | 2.55 | 4649 | <0.001 |  | 4 | 6.33 | 1.58 | 127.4 | <0.001 |  | 4 | 7.73 | 1.93 | 165.4 | <0.001 |
| Residuals | 10 | 0.005 | 0.0005 |  |  |  | 10 | 0.12 | 0.01 |  |  |  | 10 | 0.12 | 0.01 |  |  |

Table S4. Tukey HSD tests for the biodiversity partitions.

|  | Net biodiversity effect | | | |  | Selection effects | | | |  | Complementarity effects | | | |
| --- | --- | --- | --- | --- | --- | --- | --- | --- | --- | --- | --- | --- | --- | --- |
| Salinity | Diff | lwr | upr | *p* |  | Diff | lwr | upr | *p* |  | Diff | lwr | upr | *p* |
| 3.5%-0.9% | -0.200 | -0.263 | -0.137 | <0.001*** |  | 0.998 | 0.699 | 1.298 | <0.001*** |  | -1.198 | -1.489 | -0.908 | <0.001*** |
| 7%-0.9% | 0.057 | -0.006 | 0.120 | 0.081 |  | 0.812 | 0.513 | 1.112 | <0.001*** |  | -0.755 | -1.046 | -0.465 | <0.001*** |
| 15%-0.9% | 0.129 | 0.066 | 0.192 | <0.001*** |  | 1.737 | 1.437 | 2.036 | <0.001*** |  | -1.632 | -1.922 | -1.341 | <0.001*** |
| 20%-0.9% | -2.045 | -2.108 | -1.982 | <0.001*** |  | 0.032 | -0.267 | 0.332 | 0.996 |  | -2.081 | -2.371 | -1.790 | <0.001*** |
| 7%-3.5% | 0.257 | 0.194 | 0.320 | <0.001*** |  | -0.186 | -0.485 | 0.114 | 0.314 |  | 0.443 | 0.153 | 0.734 | <0.01** |
| 15%-3.5% | 0.330 | 0.267 | 0.392 | <0.001*** |  | 0.739 | 0.439 | 1.038 | <0.001*** |  | -0.433 | -0.724 | -0.143 | <0.01** |
| 20%-3.5% | -1.845 | -1.908 | -1.782 | <0.001*** |  | -0.966 | -1.265 | -0.666 | <0.001*** |  | -0.882 | -1.173 | -0.592 | <0.001*** |
| 15%-7% | 0.072 | 0.009 | 0.135 | <0.05* |  | 0.924 | 0.625 | 1.224 | <0.001*** |  | -0.876 | -1.167 | -0.586 | <0.001*** |
| 20%-7% | -2.102 | -2.165 | -2.039 | <0.001*** |  | -0.780 | -1.079 | -0.480 | <0.001*** |  | -1.325 | -1.616 | -1.035 | <0.001*** |
| 15%-20% | -2.175 | -2.237 | -2.112 | <0.001*** |  | -1.704 | -2.004 | -1.405 | <0.001*** |  | -0.449 | -0.739 | -0.158 | <0.01** |

Note: Grey shading indicates no significant difference in pairwise groups.

Table S5. The detailed composition and formulation of the enrichment media employed for the cultivation work in the sediment samples of six Qinghai lakes. The final salinity and pH of the media were adjusted to be consistent with the lake sediment samples.

| Media ID | Recipe (/L) |
| --- | --- |
| 1# | NaCl, 0.2 g; glucose, 0.5 g; tryptone, 0.01 g; 10 mL of (Na_2_SO_4_ 10H_2_O, 0.02 g; MgSO_4_ 7H_2_O, 0.02 g; KBr, 0.02 g; K_2_HPO_4_ 0.01 g; KH_2_PO_4_, 0.01 g; CaCl_2_, 0.02 g; NaHCO_3_, 0.02g; KNO_3_, 0.01 g; H_2_O, 100 mL) |
| 2# | NaCl, 0.2 g; yeast extract, 0.5 g; tryptone, 0.01 g; 10 mL of (Na_2_SO_4_ 10H_2_O, 0.02 g; MgSO_4_ 7H_2_O, 0.02 g; KBr, 0.02 g; K_2_HPO_4_ 0.01 g; KH_2_PO_4_, 0.01 g; CaCl_2_, 0.02 g; NaHCO_3_, 0.02g; KNO_3_, 0.01 g; H_2_O, 100 mL) |
| 3# | Na_2_SO_4_ 10H_2_O, 0.01 g; K_2_HPO_4_, 0.01 g; CaCO_3_, 0.002 g; KCl, 0.02 g; FeSO_4_, 0.002 g; NaCl, 0. 2 g; KNO_3_, 0.02 g, MgSO_4_.7H_2_O, 0.005 g; NaF, 0.001 g, KBr, 0.1 g, H_3_BO_3_, 0.002 g; peptone, 0.1 g |
| 4# | NaHCO_3_, 0.005 g; MgCl_2_, 0.05 g; CaCl_2_, 0.01 g, ZnSO_4_.7H_2_O, 0.001 g; FeCl_3_.6H_2_O, 0.003 g; MnCl_2_, 0.001 g; CaCl_2_, 0.002 g; Na_2_SO_4_, 10H_2_O 0.01 g; KBr, 0.1 g, MgCl_2_, 0.005 g; yeast extract, 1 g; casein acids hydrolysate, 0.75 g |
| 5# | KNO_3_, 2 g; MgSO_4_.7H_2_O, 0.05 g; K_2_HPO_4_, 2 g; CaCl_2_, 1 g; FeSO_4_, 10 mg; glucose, 10 g; casein acids hydrolysate, 0.3 g |
| 6# | NaCl, 2.0 g; KCl, 3.0 g; K_2_HPO_4_, 1.0 g; KNO_3_, 1.0 g; MgCl_2_, 5.0 g; MnCl_2_·4H_2_O, 0.02g; ZnSO_4_, 0.07 g; FeSO_4_·7H_2_O, 0.02 g; glycerol, 5.0 g; fucose, 5.0 g; asparagine, 0.5 g; vitamin B1, 0.2 mg; inositol, 0.5 mg; vitamin C, 0.2 mg |
| 7# | 1/10 of ASW medium (Na_2_SO_4_ 4.0 g, KCl 0.68 g; KBr 0.1 g, H_3_BO_3_ 0.025 g, MgCl_2_ 5.4 g; CaCl_2_.2H_2_O 1.5 g; SrCl_2_.6H_2_O 0.024 g; NaHCO_3_ 0.2 g; Na_2_HPO_4_ 0.04 g; NH_4_Cl_2_ 0.5 g; NaF 0.002 g; peptone 5.0 g, yeast extract 1.0 g) |
| 8# | NaCl, 28.13 g; KCl, 0.77 g; CaCl_2_.2H_2_O, 1.6 g; MgCl_2_.6H_2_O, 4.8 g; NaHCO_3_, 0.11 g; MgSO_4_.7H_2_O, 3.5 g |

Table S6. List of bacterial strains employed in the artificial microbial communities of this study.

| Isolate ID | Phylum | Order | Family | Genus | Lake | GenBank Accession number |
| --- | --- | --- | --- | --- | --- | --- |
| (1) CUG 91375 | *Proteobacteria* | *Pseudomonadales* | *Pseudomonadaceae* | *Pseudomonas* | QHL | OP804654 |
| (2) CUG 91386 | *Proteobacteria* | *Rhodobacterales* | *Rhodobacteraceae* | *Gemmobacter* | QHL | OP804655 |
| (3) CUG 91364 | *Proteobacteria* | *Rhizobiales* | *Phyllobacteriaceae* | *Hoeflea* | QHL | OP804656 |
| (4) CUG 91299 | *Actinomycetia* | *Micrococcales* | *Microbacteriaceae* | *Microcella* | QHL | OP804657 |
| (5) CUG 91373 | *Bacteroidetes* | *Cytophagales* | *Cyclobacteriaceae* | *Algoriphagus* | QHL | OP804658 |
| (6) CUG 91810 | *Firmicutes* | *Bacillales* | *Staphylococcaceae* | *Staphylococcus* | EHL | OP804659 |
| (7) CUG 91316 | *Proteobacteria* | *Alteromonadales* | *Marinobacter_*f | *Marinobacter* | QHL | OP804660 |
| (8) CUG 91434 | *Firmicutes* | *Bacillales* | *Paenibacillaceae* | *Paenibacillus* | EHL | OP804661 |
| (9) CUG 91328 | *Bacteroidetes* | *Cytophagales* | *Cyclobacteriaceae* | *Belliella* | QHL | OP804662 |
| (10) CUG 91887 | *Bacteroidetes* | *Cytophagales* | *Cyclobacteriaceae* | *Cyclobacterium* | EHL | OP805340 |
| (11) CUG 91570 | *Actinomycetia* | *Micrococcales* | *Micrococcaceae* | *Citricoccus* | TSL | OP804663 |
| (12) CUG 91521 | *Proteobacteria* | *Pseudomonadales* | *Pseudomonadaceae* | *Pseudomonas* | QHL | OP804664 |
| (13) CUG 91163 | *Firmicutes* | *Bacillales* | *Planococcaceae* | *Planococcus* | GHL | OP804665 |
| (14) CUG 91148 | *Firmicutes* | *Bacillales* | *Bacillaceae* | *Fictibacillus* | GHL | OP804666 |
| (15) CUG 91061 | *Firmicutes* | *Bacillales* | *Planococcaceae* | *Planomicrobium* | GHL | OP804667 |
| (16) CUG 91802 | *Actinomycetia* | *Micrococcales* | *Micrococcaceae* | *Kocuria* | EHL | OP804668 |
| (17) CUG 91232 | *Actinomycetia* | *Corynebacteriales* | *Dietziaceae* | *Dietzia* | TSL | OP804669 |
| (18) CUG 91106 | *Bacteroidetes* | *Flavobacteriales* | *Flavobacteriaceae* | *Salegentibacter* | GHL | OP804670 |
| (19) CUG 91150 | *Actinomycetia* | *Micrococcales* | *Promicromonosporaceae* | *Isoptericola* | GHL | OP804671 |
| (20) CUG 91508 | *Proteobacteria* | *Alteromonadales* | *Idiomarinaceae* | *Idiomarina* | XCDL | OP804672 |
| (21) CUG 91730 | *Proteobacteria* | *Alteromonadales* | *Marinobacter_*f | *Marinobacter* | QHL | OP804673 |
| (22) CUG 91204 | *Bacteroidetes* | *Flavobacteriales* | *Flavobacteriaceae* | *Salegentibacter* | XCDL | OP804674 |
| (23) CUG 91544 | *Firmicutes* | *Bacillales* | *Planococcaceae* | *Jeotgalibacillus* | CKL | OP804675 |
| (24) CUG 91736 | *Firmicutes* | *Bacillales* | *Bacillaceae* | *Bacillus* | QHL | OP804676 |
| (25) CUG 91563 | *Firmicutes* | *Bacillales* | *Bacillaceae* | *Gracilibacillus* | TSL | OP804677 |
| (26) CUG 91487 | *Proteobacteria* | *Alteromonadales* | *Marinobacter_*f | *Marinobacter* | GHL | OP804678 |
| (27) CUG 91531 | *Proteobacteria* | *Oceanospirillales* | *Halomonadaceae* | *Halomonas* | TSL | OP804679 |
| (28) CUG 91013 | *Firmicutes* | *Bacillales* | *Planococcaceae* | *Planococcus* | GHL | OP804680 |
| (29) CUG 91523 | *Firmicutes* | *Bacillales* | *Bacillaceae* | *Thalassobacillus* | TSL | OP804681 |
| (30) CUG 91494 | *Firmicutes* | *Bacillales* | *Bacillaceae* | *Virgibacillus* | CKL | OP804682 |
| (31) CUG 91551 | *Firmicutes* | *Bacillales* | *Staphylococcaceae* | *Staphylococcus* | XCDL | OP804683 |
| (32) CUG 91578 | *Actinomycetia* | *Micrococcales* | *Micrococcaceae* | *Nesterenkonia* | GHL | OP804684 |
| (33) CUG 91574 | *Firmicutes* | *Bacillales* | *Staphylococcaceae* | *Salinicoccus* | GHL | OP804685 |
| (34) CUG 91527 | *Proteobacteria* | *Oceanospirillales* | *Halomonadaceae* | *Halomonas* | TSL | OP804686 |
| (35) CUG 91135 | *Firmicutes* | *Bacillales* | *Bacillaceae* | *Halobacillus* | XCDL | OP804687 |
| (36) CUG 91119 | *Firmicutes* | *Bacillales* | *Bacillaceae* | *Marinococcus* | XCDL | OP804688 |
| (37) CUG 92003 | *Firmicutes* | *Bacillales* | *Bacillaceae* | *Thalassobacillus* | GHL | OP804689 |
| (38) CUG 91675 | *Firmicutes* | *Bacillales* | *Bacillaceae* | *Halolactibacillus* | GHL | OP804690 |
| (39) CUG 91138 | *Firmicutes* | *Bacillales* | *Bacillaceae* | *Oceanobacillus* | XCDL | OP804691 |
| (40) CUG 91694 | *Firmicutes* | *Bacillales* | *Bacillaceae* | *Terribacillus* | GHL | OP804692 |

Note: EHL: Erhai Lake, QHL: Qinghai Lake, TSL: Tuosu Lake, GHL: Gahai Lake, XCDL: Xiaochaidan Lake, CKL: Chaka Lake. The number in parentheses is the identity of each isolated taxa.

Table S7. Experimental design of the artificial microbial communities with different levels of species richness.

| Richness | Replicate | | Number | Vol (ml) |
| --- | --- | --- | --- | --- |
| Sp.5 | A1 | | 5, 14, 21, 25, 36 | 8 |
| Sp.5 | A2 | | 4, 10, 17, 28, 33 | 8 |
| Sp.5 | A3 | | 6, 11, 20, 27, 40 | 8 |
| Sp.5 | A4 | | 8, 16, 18, 32, 34 | 8 |
| Sp.10 | B1 | | 5, 14, 21, 25, 36, 4, 10, 17, 28, 33 | 4 |
| Sp.10 | B2 | | 6, 11, 20, 27, 40, 8, 16, 18, 32, 34 | 4 |
| Sp.10 | B3 | | 8, 16, 21, 26, 35, 2, 15, 22, 32, 38 | 4 |
| Sp.10 | B4 | | 4, 14, 23, 28, 36, 5, 12, 24, 29, 39 | 4 |
| Sp.10 | B5 | | 3, 9, 17, 32, 36, 4, 15, 24, 31, 40 | 4 |
| Sp.10 | B6 | | 7, 14, 18, 32, 37, 1, 10, 23, 30, 33 | 4 |
| Sp.10 | B7 | | 4, 11, 20, 29, 38, 6, 13, 21, 30, 34 | 4 |
| Sp.10 | B8 | | 7, 12, 22, 32, 39, 2, 10, 19, 28, 35 | 4 |
| Sp.20 | C1 | | 1, 16, 17, 25, 33, 2, 10, 18, 26, 34, 3, 11, 19, 27, 35, 4, 12, 20, 28, 36 | 2 |
| Sp.20 | C2 | | 1, 9, 17, 25, 33, 2, 10, 18, 26, 34, 3, 11, 19, 27, 35, 4, 12, 20, 28, 36 | 2 |
| Sp.20 | C3 | | 5, 13, 21, 29, 37, 6, 14, 22, 30, 38, 7, 15, 23, 31, 39, 8, 16, 24, 32, 40 | 2 |
| Sp.20 | C4 | | 3, 9, 17, 32, 36, 4, 15, 24, 31, 40, 7, 14, 18, 32, 37, 1, 10, 23, 30, 33 | 2 |
| Sp.20 | C5 | | 4, 11, 20, 29, 38, 6, 13, 21, 30, 34, 7, 12, 22, 32, 39, 2, 10, 19, 28, 35 | 2 |
| Sp.20 | C6 | | 3, 11, 19, 27, 35, 4, 12, 20, 28, 36, 7, 15, 23, 31, 38, 8, 16, 24, 32, 40 | 2 |
| Sp.20 | C7 | | 3, 11, 19, 27, 35, 4, 12, 20, 28, 36, 6, 14, 22, 30, 38, 1, 9, 17, 25, 39 | 2 |
| Sp.20 | C8 | | 2, 10, 18, 26, 34, 1, 11, 19, 27, 37, 6, 14, 22, 30, 33, 7, 15, 21, 31, 39 | 2 |
| Sp.20 | C9 | | 4, 12, 20, 28, 36, 6, 14, 22, 30, 37, 7, 15, 23, 31, 39, 8, 16, 24, 32, 40 | 2 |
| Sp.20 | C10 | | 2, 10, 18, 26, 34, 3, 11, 19, 27, 35, 4, 12, 20, 28, 36, 5, 13, 21, 29, 37 | 2 |
| Sp.20 | C11 | | 1, 9, 17, 25, 33, 2, 10, 18, 26, 34, 5, 13, 21, 29, 37, 6, 14, 22, 30, 38 | 2 |
| Sp.20 | | C12 | 7, 15, 23, 31, 39, 8, 16, 24, 32, 40, 3, 11, 19, 27, 35, 5, 13, 21, 29, 37 | 2 |
| Sp.20 | C13 | | 1, 9, 17, 25, 33, 2, 10, 18, 26, 34, 3, 11, 19, 27, 35, 5, 13, 22, 29, 37 | 2 |
| Sp.20 | C14 | | 2, 9, 20, 25, 37, 3, 10, 21, 26, 38, 4, 11, 22, 27, 39, 5, 12, 23, 28, 40 | 2 |
| Sp.20 | C15 | | 5, 9, 21, 25, 37, 6, 10, 22, 26, 36, 7, 11, 23, 27, 39, 8, 12, 24, 28, 40 | 2 |
| Sp.20 | C16 | | 4, 10, 20, 25, 35, 5, 11, 21, 26, 36, 6, 12, 22, 27, 37, 7, 13, 23, 28, 39 | 2 |
| Sp.40 | D1 | | All 40 species | 1 |
| Sp.40 | D2 | | All 40 species | 1 |
| Sp.40 | D3 | | All 40 species | 1 |

Note: Richness represents the number of species assembled. Numbers indicate the identity of each isolate assembled into each artificial community (see Table S6 for detailed information on the bacterial isolates), and volume describes the media volume for each isolate added to each of the artificial communities.
